# Editorial: Immune Outposts on the Inflammatory Frontier: Tertiary Lymphoid Structures as Targets for Immunotherapy of Cancer and Autoimmunity

**DOI:** 10.3389/fimmu.2019.00993

**Published:** 2019-05-03

**Authors:** Karina Silina, David Kroeger

**Affiliations:** ^1^Institute of Experimental Immunology, University of Zurich, Zurich, Switzerland; ^2^Formerly Deeley Research Centre, British Columbia Cancer Agency, Victoria, BC, Canada

**Keywords:** tertiary lymphoid structures, tertiary lymphoid organs, ectopic germinal centers, autoimmunity, transplantation, cancer microenvironment

Tertiary lymphoid structures (TLS), also known as tertiary or ectopic lymphoid organs, develop in chronically inflamed tissues via the process termed lymphoid neogenesis (1). This process follows similar molecular queues as lymphoid organogenesis during embryonic development (2). TLS have long been known in the field of autoimmune diseases and transplantation as potential sites of auto- and alloreactive lymphocyte activation contributing to disease exacerbation, resistance to therapy and transplant rejection (3, 4). By contrast, TLS correlate with improved outcome of cancer patients, an observation that has been reproduced in several different tumor types (5–8). The data from models of infection and inflammation provide evidence that TLS effectively replicate the functions of secondary lymphoid organs (4, 9), supporting the hypothesis that deliberate manipulation of lymphoid neogenesis might offer novel therapeutic opportunities to control autoimmunity and cancer (4, 7). However, there are still many gaps in our understanding of TLS biology. For example, not all solid tumor types seem to exhibit a positive correlation between TLS development and survival. TLS in hepatocellular carcinoma are proposed to nurse tumor progenitor cells and correlate with increased tumor initiation (10). Furthermore, in different models of inflammation TLS provide tolerogenic rather than potentiating effects (7). Which inflammatory stimuli dictate lymphoid neogenesis in various organs and how TLS functions regulated are important questions that need to be addressed before rational strategies for therapeutic manipulation of TLS can be developed. In this special topic, we are pleased to offer readers the latest insights into these and other critical questions in the field.

Colbeck et al. provide a comprehensive overview of TLS development, prognostic relevance and a variety of functional effects in cancer in their review article *Tertiary Lymphoid Structures in Cancer: Drivers of Antitumor Immunity, immunosuppression, or Bystander Sentinels in Disease?* The authors also discuss potential novel therapeutic strategies to manipulate TLS development including targeting the TNFR pathway. In a similar vein, the review from Teillaud and Dieu-Nosjean “*Tertiary Lymphoid Structures: an Anti-tumor School for Adaptive Immune Cells and an Antibody Factory to Fight Cancer?*” describes in detail the latest data on the role of TLS in generating antigen-specific humoral and cellular immunity in various tumors. Here the therapeutic potential of TLS in the context of various immunotherapies is discussed.

In breast cancer, immune checkpoint inhibition has not met the same clinical success as in melanoma and lung cancer patients (11). The original work from Solinas et al. *Immune Checkpoint Molecules on Tumor-Infiltrating Lymphocytes and Their Association with Tertiary Lymphoid Structures in Human Breast Cancer* explores the phenotype of breast cancer infiltrating T cells and reveals a positive correlation between PD-1^+^ T cells in tumor-associated TLS and patient survival. The results of gene expression profiling in this study show that increased checkpoint molecule expression is correlated with the extent of T cell infiltration and TLS development and could help guide patient selection for immunotherapy.

In contrast to findings in other solid tumor types, B cell- and TLS-associated molecules have been correlated to tumor progression in prostate cancer (12). Here we are happy to present the first direct analysis of TLS in prostate cancer reported by the lab of Rangel-Moreno in their original research article “*A Unique Cellular and Molecular Microenvironment is Present in Tertiary Lymphoid Organs of Patients With Spontaneous Prostate Cancer Regression*.” Their results demonstrate that TLS possess a unique composition in regressing prostate tumors. The authors also provide new insights into possible novel immunotherapeutic strategies for prostate cancer by targeting COX2 and T regulatory cells.

The perspective by Zhu et al. entitled *Tumor-Associated Tertiary Lymphoid structures: Gene-Expression Profiling and their Bioengineering* deals with the possible technological solutions to aid lymphoid neogenesis in cancer using scaffold materials in combination with chemokine queues or relevant cellular components, such as dendritic cells. The authors also review the potential to use gene expression signatures that identify tumors with extensive lymphoid neogenesis as a patient stratification approach for immunotherapy.

While TLS induction, and consequent lymphocyte activation, would appear to be favorable in cancer, the inhibition of lymphoid neogenesis might represent a means to limit autoimmunity and transplant rejection. Alsughayyir et al. discuss the differences in regulatory circuits in secondary lymphoid organs and TLS, especially regarding the germinal center reaction, that might explain the contribution of TLS to autoimmune pathologies in their review article *Spoiling for a Fight: B Lymphocytes As initiator and effector Populations within Tertiary Lymphoid Organs in Autoimmunity and Transplantation*. The authors also provide a detailed overview of the mechanisms of TLS development in autoimmune conditions and transplantation.

The original work form the group of Le Panse in Paris addresses a particularly relevant topic—the development of adequate experimental models for studying TLS function in autoimmunity. The original study *Use of Toll-like Receptor Agonists to Induce Ectopic Lymphoid Structures in Myasthenia Gravis Mouse Models* describes the effects of various inflammatory stimuli on the development of TLS and germinal centers in the murine thymus, a characteristic feature of myasthenia gravis in humans. The authors highlight the complexity of Toll-like receptor signaling in lymphoid neogenesis in different peripheral organs and indicate toward potential differences between humans and mice.

In summary, our research topic has brought together a collection of reviews and original articles providing the latest insights and opinions in the field of lymphoid neogenesis. We hope all readers will enjoy learning more about TLS and their potential therapeutic relevance in cancer and immune pathologies.

## Author Contributions

All authors listed have made a substantial, direct and intellectual contribution to the work, and approved it for publication.

### Conflict of Interest Statement

The authors declare that the research was conducted in the absence of any commercial or financial relationships that could be construed as a potential conflict of interest.
